# A simple approach to assess the cancer risk of occupational exposure to genotoxic drugs in healthcare settings

**DOI:** 10.1186/s12995-022-00349-z

**Published:** 2022-04-01

**Authors:** Johannes Gerding, Lea Anhäuser, Udo Eickmann, Albert Nienhaus

**Affiliations:** 1 Department for Occupational Medicine, Hazardous Substances and Public Health, German Social Accident Insurance, Institution for the Health and Welfare Services (BGW), Pappelallee 33/35/37, 22089 Hamburg, Germany; 2 Competence Centre for Epidemiology and Health Services Research for Healthcare Professionals (CVcare), University Medical Centre Hamburg-Eppendorf (UKE), Martinistraße 52, 20246 Hamburg, Germany

**Keywords:** Occupational exposure, Health workers, Genotoxic drugs, Risk assessment, Cancer, Threshold of toxicological concern

## Abstract

**Background:**

Several drugs for human use possess genotoxic properties as a necessary consequence of their intended therapeutic effect (e.g. antineoplastics). Health workers may be exposed to these chemicals in various occupational settings such as dose preparation and administration. To date, there are no quantitative risk assessment models to estimate the cancer risk of health workers due to the handling of genotoxic drugs. We therefore developed a quantitative risk assessment model to assess the cancer risk of occupational exposure to genotoxic drugs in healthcare settings based on the threshold of toxicological concern (TTC) concept. This model was used to evaluate the cancer risk of health workers due to the handling of genotoxic drugs in modern health care facilities.

**Methods:**

We modified the threshold of toxicological concern (TTC) concept to fit the purpose of occupational cancer risk assessment. The risk model underlying ICH guideline M7 (R1): “assessment and control of DNA reactive (mutagenic) impurities in pharmaceuticals to limit potential carcinogenic risk” was used as a starting point for our model. We conducted a short review of studies on the occupational exposure of health workers to genotoxic drugs. These occupational exposure data were compared to the acceptable exposure levels resulting from our TTC based risk model.

**Results:**

Based on the threshold of toxicological concern (TTC) concept, we defined an acceptable daily intake (ADI) of 4 μg/day as threshold of no concern for the exposure of health workers to genotoxic drugs. Regarding the dermal exposure of health workers to genotoxic drugs, we derived a corresponding acceptable surface contamination level (ASCL) of 20 ng/cm^2^. Both ADI and ASCL are usually not exceeded in modern healthcare settings. Current safety precautions provide sufficient protection to health workers.

**Conclusions:**

The application of our model indicates that workers in modern healthcare facilities are not at risk of developing work related cancer above widely accepted cancer risk levels due to the occupational exposure to genotoxic drugs. Hence, the present study may assist employers and public authorities to make informed decisions concerning the need for (further) protective measures and during risk communication to health workers.

## Background

Antineoplastic drugs play a vivid role in modern cancer treatment. As a necessary consequence of their intended therapeutic effect (e.g. inhibiting tumour growth), many of the active pharmaceutical ingredients (APIs) of these drugs are carcinogenic, mutagenic or toxic to reproduction in humans at therapeutic doses [1]. The hazardous properties of antineoplastic drugs are accepted in favour of their indispensable value for the treatment of cancer, whereas occupational exposure of health workers to these substances has to be controlled in order to prevent work-related diseases. Hazardous drugs are handled in various settings in healthcare facilities such as preparing doses in the pharmacy or drug administration on the hospital ward [2]. Healthcare providers usually do not have access to adequate toxicity data nor to trained staff setting and controlling company-based occupational exposure limits. In contrast to workplaces in the pharmaceutical industry, risk assessment strategies in healthcare settings have to consider the parallel handling of various hazardous drugs in a less controlled environment. To prevent harmful exposure to hazardous drugs, several institutions issued guidelines for the safe handling of hazardous drugs in healthcare settings. This has led to a high level of protective measures to ensure occupational safety across many countries and regions [3]. The regular use of biologic safety cabinets (BSC), closed system drug transfer systems (CSTD) and personal protective equipment (PPE) has reduced the actual exposure of health workers [4]. Anyhow, existing approaches to assess the health risk associated with the occupational exposure towards drugs are hazard based and aim on reducing the workers exposure as low as (reasonably) possible [4–6]. To date, there are no quantitative measures to identify occupational (low dose) exposures, that put health workers at risk to develop a work-related disease or not. Therefore, no safe levels of inhalative or dermal exposure have been set yet for non-threshold genotoxic (mutagenic) drugs. The lack of (controllable) occupational exposure limits constantly raises the question, if the current occupational safety precautions are sufficient or if higher standards need to be established [7, 8]. This demonstrates the need for risk assessment models that are able to define safe working conditions for health workers handling hazardous drugs based on reliable toxicity data. In this study, we applied the threshold of toxicological concern (TTC) concept to assess the cancer risk of health workers from exposures to genotoxic drugs that occur in modern healthcare facilities. The TTC concept has successfully been applied to assess the health risks arising from undesired genotoxic food and drug contaminants as well as for setting occupational exposure limits for genotoxic substances in the pharmaceutical industry [9–11]. Its applicability to the assessment and control of cancer risks resulting from the occupational exposure to genotoxic drugs in healthcare settings was evaluated in the present study.

## Methods

The International Council for Harmonisation of Technical Requirements for Registration of Pharmaceuticals for Human Use (ICH) used the TCC concept in the guideline M7 (R1): “Assessment and control of DNA reactive (mutagenic) impurities in pharmaceuticals to limit potential carcinogenic risk”, that aims on ensuring patients safety during long term drug treatments [10]. We discuss the TTC concept in the light of this approach and adapted the ICH approach to derive acceptable threshold values of exposure by calculating an acceptable daily intake (ADI) of genotoxic drugs for health workers. Certain substances are exempt from the application of the TTC-approach by definition. The TTC-concept cannot be applied to high potency carcinogens (aflatoxin-like compounds, *N*-nitroso-compounds and azoxy-compounds). We therefore reviewed the chemical structures of the antineoplastic drugs listed in the current National Institute for Occupational Safety and Health (NIOSH) list of antineoplastic and other hazardous drugs for the presence of the structural alerts mentioned above [12] (“Using the TTC concept to define acceptable levels of exposures to genotoxic drugs in healthcare settings” section).

In a second step, we reviewed publications on the occupational exposure of health workers in modern healthcare facilities. For this purpose, we used the PubMed database (date of research: 30th April 2020) applying the search terms “hazardous drugs and occupational exposure and surface contamination” or “hazardous drugs and occupational exposure and dermal exposure” or “hazardous drugs and occupational exposure and inhalation”. The literature search was limited to publication dates from 2000 until the date of research (“Occupational exposure to hazardous drugs in healthcare settings” section). The dermal exposure of health workers to hazardous drugs was subsequently quantified as daily dermal intake (DDI) using literature data and the model from Kimmel and co-workers [13] (“Quantifying the exposure of health workers to genotoxic drugs” section). In a final risk assessment step, we calculated an acceptable surface contamination level (ASCL) as an indicator of occupational drug exposure and compared it with the exposure data found in literature (“Proposal of an acceptable surface contamination level and risk assessment” section).

## Results

### Using the TTC concept to define acceptable levels of exposures to genotoxic drugs in healthcare settings

The TTC concept was originally proposed to assess the risk of human exposure to poorly investigated chemicals and to define acceptable limits of intake for such compounds [14–16]. During the last decades, the TTC concept was frequently modified to cover various toxicological endpoints including (genotoxic) carcinogenicity [11]. Regarding this endpoint, the TTC concept is based on a large dataset of TD_50_ values (doses resulting in a 50% tumour incidence in animal studies) of known carcinogens. Using a simple linear extrapolation, Kroes et al. defined intakes that correspond to an excess lifetime cancer risk of 1 additional cancer case in 1.000.000 exposed individuals [17]. For the vast majority of compounds the acceptable daily intake was set to 0.15 μg/day for the lifetime exposure to a poorly investigated chemical. Certain high potency genotoxic carcinogens such as aflatoxin-like compounds, *N*-nitroso-compounds and azoxy-compounds were exempt from the scope of the TTC concept, since they are expected to show carcinogenic effects at or below a daily intake of 0.15 μg/day [17]. Other compounds not covered by the TTC-approach are metals and proteins, that were not included in the initial database to calculate the TTC by Munro (1996) as well as highly bioaccumulative compounds such as polyhalogenated dibenzo-p-dioxins,-dibenzofurans and –biphenyls [15, 17]. We therefore reviewed the chemical structure of 116 common antineoplastic drugs listed in Table 1 of the current NIOSH list of antineoplastic and other hazardous drugs for the presence of exclusion criteria [12]. Ten drug molecules contain metals (arsenic, platinum) or proteins ((conjugated) monoclonal antibodies) and are not covered by the TTC-approach. Seven of the remaining drug molecules were classified as carcinogens (Cat. 1) and 13 as carcinogens (Cat. 2A/B) by the International Agency for Research on Cancer (IARC). A group of three molecules (carmustine, lomustine and streptocin) triggered the structural alert for high potency carcinogenicity (*N*-nitroso-compounds). The TTC-based risk assessment cannot be applied to these substances. In conclusion, the TTC-concept is generally applicable to the vast majority antineoplastic drugs with the exclusion of metals and proteins and after careful consideration of structural alerts for high potency carcinogenicity. Today, the TTC concept is widely accepted and applied in various regulatory settings such as food and drug safety [10, 38]. In 2018, the European Medicines Agency (EMA) published the latest version of an ICH-guideline on the assessment and control of genotoxic impurities in drugs. The ICH M7 (R1) guideline defines safe maximum levels for undesired genotoxic impurities in drugs to prevent patients from suffering treatment-associated cancer. Based on the linear extrapolation of the TTC concept, the ICH M7 guideline defines a tenfold increased maximum intake of 1.5 μg per day accepting one additional cancer case in 100.000 patients at daily lifetime exposure (70 years) to a genotoxic drug impurity. The excess lifetime cancer risk of 1 additional cancer case per 100.000 treated patients was accepted in favour of the health benefit resulting from the treatment. Based on these assumptions, ICH calculated acceptable daily intakes (ADI) for individual drug impurities for different treatment durations from less than 1 month (ADI 120 μg/day) to lifetime exposure (1.5 μg/day). For multiple impurities, the ADI for lifetime exposure was set to 5 μg/day. In summary, the ICH approach addresses the problem of long-term low dose exposures to genotoxic chemicals based on a very conservative risk model using simple linear extrapolation to different exposure scenarios.

**Table 1:** Review on hazardous drug contamination of surfaces in healthcare settings. Analytes include *5-FU* 5-fluorouracil, *CP* Cyclophosphamide, *DX* Docetaxel, *GM* Gemcitabine, *IF* Ifosphamide, *MT* Methotrexate, *PX* Paclitaxel, *Pt* Platinum compounds. Note: Pt-compound are not covered by the TTC-concept

Facility	Substances	Sample Number	Surface contamination	Source	Remark
**Pharmacy**	CP	114	0.0027–0.0055 pg/cm^2^	[18]	closed system drug transfer device
	CP, IF, MT	219	ND-0.751 ng/cm^2^ (median)	[19]	after refitting laboratory (but also new technicians)
	CP	6	65 ng/cm^2^ (57–110 ng/cm^2^)	[20]	high cleaning efficacy
	CP, IF, 5-FU	264	0.03–186.8 ng/cm^2^	[21]	use of CSTD, means below 2 ng/cm^2^
	CP	75	< 0.0015–11 ng/cm^2^	[22]	no use of CSTD, means below 0.02 ng/cm^2^
	CP, IF, GM	109	<LOD-17.4 ng/cm^2^ (mean)	[23]	
	CP, Pt	50	5–368 pg/cm^2^	[24]	protective measures (contamination and cleaning)
	CP	152	3.91–153 pg/cm^2^ (75% percentile)	[25]	annual setting of goals for surface contamination
	CP	193	4.3–81.5 pg/cm^2^ (75% percentile)	[26]	
	CP, 5-FU	104	0.002–0.018 ng/cm^2^ (median)	[27]	before use of CSTD
	CP	60	0.01–0.06 ng/cm^2^ (median)	[28]	use of CSTD for 1 and 8 months
	CP	248	0.005–0.087 ng/cm^2^ (75% percentile)	[29]	traces of contamination
	PX, DX, IF, CP, 5-FU	1541	> 1.08 ng/cm^2^	[30]	other positive samples were < 1.08 ng/cm^2^ to ≤0.0108 ng/cm^2^ (nd)
**Hospital -**	CP, Pt	50	< 0.2–371 pg/cm^2^	[24]	protective measures (contamination and cleaning)
Administration	CP, 5-FU, PX	186	0.7–21 μg/cm^2^	[31]	authors report insufficient protocols for cleaning
	CP, 5-FU	104	0.007–0.019 ng/cm^2^ (median)	[27]	before use of CSTD
	CP, IP, GM, 5-FU	19,479	0.8–236,097 pg/cm^2^	[5]	development of a monitoring protocol
	CP, 5-FU	50	0.2–270.24 μg/cm^2^	[32]	authors report insufficient protocols for cleaning
	CP, 5-FU, Pt	120	<LOD-181,800 pg/cm^2^	[33]	personal protective equipment, environmental monitoring
**Hospital -**	CP	56	< 0.0015–28 ng/cm^2^	[22]	no use of CSTD, means below 0.02 ng/cm^2^
Patient care	CP, IF	724	0.43–23 pg/cm^2^ (median)	[34]	
area	CP, IP	60	0.03–0.15 ng/cm^2^ (median)	[35]	tube priming in pharmacy
	CP	143	4.77–159 pg/cm^2^ (75% percentile)	[25]	
	CP	189	3.5–91.0 pg/cm^2^ (75% percentile)	[26]	environmental surveillance
	CP	238	0.0017–0.065 ng/cm^2^ (75% percentile)	[29]	
**Hospital -**	Pt	52	0.22–110,000 pg/cm^2^	[36]	means below 5.4 pg/cm^2^
Operating room	Pt	168	0.1–1733 pg/cm^2^ (median 0.06–9.42 pg/cm^2^)	[37]	safety and cleaning standards, regulatory monitoring

In contrast to patients, (health) workers do not experience health benefits from the exposure to hazardous drugs. However, it is widely accepted that the workplace is not a zero-risk environment. For example, in German occupational safety and health legislation, occupational exposure to a carcinogenic chemical is accepted up to an excess lifetime cancer risk of 4 additional cancer case in 100.000 workers (see Technical Rule for Hazardous Substances (TRGS) 910) [39]. This acceptable risk is considerably lower than the decision point for an ‘acceptable’ lifetime cancer risk of 1 additional cancer case in 10.000 workers in the European Union and other regions [40]. Risk-related occupational exposure limits can be derived, when sufficient toxicity data are available [39]. In the absence of sound toxicity data, however, it seems feasible to transfer the patient safety oriented ICH risk assessment approach to the evaluation of cancer risks associated with the occupational exposure of health workers to genotoxic drugs.

As the TTC concept is based on a linear extrapolation of cancer risks, it can easily be adjusted to different settings. We take an ADI of 1.5 μg/day (excess lifetime cancer risk of 1:100.000) from the ICH approach as point of departure (POD) for an acceptabe level of exposure. An ADI of 1.5 μg/day corresponds to a maximum lifetime intake (MLI) of 38,325 μg of genotoxic drug contaminants assuming daily exposure during 70 Years (25,550 days of exposure). Occupational exposure of healthcare staff handling genotoxic drugs usually takes place during a shorter timeframe (usually less than 40 Years). Consequently, the ADI can be adjusted using Formula 1:

1 $$ ADI\ \left(\mu g/ day\right)=\frac{MLI\ \left(\mu g\right)}{t_{Ex}\ (days)} $$

With ADI = Acceptable daily intake (μg/day)

MLI = Maximum lifetime intake (μg)

t_Ex_ = Lifetime exposure days

Assuming an average working life of 40 years (240 exposure days/year) the ADI for drug handling health workers can be set to 4 μg/day without changing the excess lifetime cancer risk of 1 additional work related cancer case in 100.000 workers. Formula 1 can be adjusted for different cancer risks e.g. to fit regional (regulatory) requirements. Using formula 2, an adjusted ADI of 16 μg/day results from the application of an acceptable lifetime excess cancer risk of 4:100.000 as stated in German occupational safety and health legislation. Anyhow, we continue our risk assessment with the conservative assumption of an ADI of 4 μg/day.

2 $$ {ADI}_{Adj}\ \left(\mu g/ day\right)=\frac{MLI\ \left(\mu g\right)}{t_{Ex}\ (days)}\times \frac{ECR_{Adj}}{ECR_{Def}} $$

With ADI_Adj_ = Acceptable daily intake (μg/day)

MLI = Maximum lifetime intake (μg)

t_Ex_ = Lifetime exposure days

ECR_Def_ = Default excess cancer risk of 1:100.000

ECR_Adj_ = Adjusted excess cancer risk e.g. 4:100.000

### Occupational exposure to hazardous drugs in healthcare settings

Health workers may be exposed to hazardous drugs performing various tasks such as unpacking, dose preparation in the pharmacy and administration on the hospital ward. Exposure to genotoxic drugs may also occur during waste handling or following contact to urine and other body fluids of patients under (high dose) therapy. The vast majority of hazardous drugs arrive at the healthcare facility as solutions ready for infusion or as powder to be reconstituted for intravenous administration. (Coated) tablets play a minor role in cancer treatment and exposure of healthcare staff to the API can be neglected as long as the tablet is administered undamaged. Drug exposure may occur by inhalation or oral intake of airborne dust and particles or through dermal contact to contaminated surfaces or devices. In modern healthcare settings, strict safe handling guidelines are in force to reduce potential exposures to hazardous drugs to a reasonable minimum [3]. Hazardous drugs are usually handled using a variety of protective measures such as drug safety cabinets for preparation, chemo spikes and gloves. It can be assumed that the use of safety cabinets prevents any inhalative or oral exposure to airborne drug dust and particles [41]. Outside of drug safety cabinets, hazardous drugs are handled as aqueous solutions only and inhalative or oral exposure is unlikely [42]. The inhalative exposure to hazardous drugs is also neglectable, when cytotoxic drugs are aerosolized on purpose to be intraperitonealy administered during surgeries [37]. Dermal exposure therefore seems to be the most relevant route of exposure of healthcare staff to hazardous drugs. It may occur, if the unprotected skin (hands) of staff gets in contact with contaminated surfaces or accidentally spilled drug solutions.

The aforementioned principle considerations are in line with the results of our recent review of scientific publications on hazardous drugs exposure in healthcare settings. A PubMed database request returned a total of 70 original publications and reviews (see methods part for details). The vast majority of studies addressed the dermal exposure and the contamination of surfaces with hazardous drugs (*n* = 37). Inhalative exposure was studied in seven publications. Twenty-six of the publications were off-topic. Quantitative data was only available for potential dermal exposure (surface contamination levels). Table 1 summarizes the results of the retrieved publications containing quantitative data.

On surfaces in the hospital pharmacy, drug administration areas, patient care areas and operation rooms, contaminations usually occurred within a range of a few pg/cm^2^ up to 200 ng/cm^2^. Mean and median concentrations were usually below 17.4 ng/cm^2^. Higher surface contaminations of up to 270 μg/cm^2^ were only reported by Viegas et al. (2014 and 2018) and Touzin et al. (2010). Viegas et al. (2014 and 2018) documented inadequate cleaning protocols and incorrect working procedures. Touzin et al. (2010) reported on the unsatisfying cleaning efficacy of a new cleaning protocol. It can therefore be concluded, that contamination of surfaces with hazardous drug in modern healthcare facilities usually does not exceed concentrations of 200 ng/cm^2^. Mean and median surface contaminations are considerably lower (few pg/cm^2^ up to 17.4 ng/cm^2^).

#### Quantifying the exposure of health workers to genotoxic drugs

Considering the results of our short review, we identified the dermal exposure as the major contributor to the total exposure of health workers to hazardous drugs. Few attempts to quantify and assess the dermal exposure of surface contaminants at the workplace have been made so far. A major obstacle in quantifying the dermal exposure is the limited knowledge on the transfer rates of (drug) molecules across the skin barrier. Conservative models therefore rely on the assumption that 100% of the applied substances are incorporated. Considering these limitations, Kimmel and co-workers developed a simple but useful model to quantify the contribution of dermal exposure to the total uptake of hazardous drugs at workplaces in the pharmaceutical industry [13]. A worst-case scenario assumes, that the daily dermal exposure equals the amount of substance on a surface area of 200 cm^2^ (skin area of both palms). Using the simple yet very conservative model of Kimmel et al. (2011) the daily dermal intake (DDI) of hazardous drugs can be derived using the following formula:

3 $$ DDI\ \left(\mu g/ day\right)=\frac{SA_{Ex}\left({cm}^2\right)\times SC\ \left(\mu g/{cm}^2\right)}{AF_{Bio}} $$

With DDI = Daily dermal intake (μg/day)

SA_Ex_ = Exposed skin area (set to 200 cm^2^ by default)

SC = Surface contamination (μg/cm^2^)

AF_Bio_ = Adjustment factor bioavailability (100% = 1)

Considering the highest mean surface contamination value reported in the reviewed studies (17.4 ng/cm^2^, see Table 1), the mean DDI of health workers may be estimated to be below 3500 ng/day (3.5 μg/day) and the thereby below the previously proposed ADI of 4 μg/day.

### Proposal of an acceptable surface contamination level and risk assessment

In “Using the TTC concept to define acceptable levels of exposures to genotoxic drugs in healthcare settings” section of this paper, we used a modified TTC-approach to estimate an acceptable daily intake (ADI) of genotoxic drugs of 4 μg/day that would not increase the risk of health workers suffering from work related cancer by 1 excess case in 100.000 workers following lifetime exposure (40 years). We subsequently identified the dermal contact to be the major route of exposure and derived the daily dermal uptake (DDI) as indicator of the total drug uptake (see “Occupational exposure to hazardous drugs in healthcare settings” section). Hence, safe working conditions can be assumed if

$$ \frac{DDI}{ADI}\le 1 $$

Since the DDI is only influenced by the extent of contamination of the surface getting in contact with the workers skin and the ADI is fixed to 4000 ng/day, an acceptable surface contamination level (ASCL) of 20 ng/cm^2^ may be derived as follows:

4 $$ ASCL\ \left(\frac{ng}{cm^2}\right)=\frac{ADI\ \left(4000\frac{ng}{day}\right)}{SA_{Ex}\ \left(200\frac{cm^2}{day}\right)}=20\  ng/{cm}^2 $$

With

ASCL = Acceptable surface contamination level (ng/cm^2^)

ADI = Acceptable daily intake

SA_Ex_ = Exposed skin area (set to 200 cm^2^ by default)

The ASCL may serve as a conservative threshold of no concern for surface contaminations to protect health workers from suffering work related cancer.

The results of the review (see Table 1) show, that health workers usually do not get in contact with surfaces, that are contaminated above the aforementioned ASCL of 20 ng/cm^2^. Peak exposures may occur, if concentrated drug solutions are spilled in larger amounts and get in contact with the unprotected skin. Such exposures occur only accidental and are limited to a few lifetime events. They are unlikely to have a disproportionate effect on systemic concentrations that would significantly increase the lifetime (dermal) drug intake of health workers. It can therefore be concluded, that following modern drug handling guidelines, health works are not at risk of developing work related cancer due to occupational exposure to hazardous (genotoxic) drugs.

## Discussion

The present study is a quantitative approach to evaluate the cancer risk of health workers due to the occupational exposure to genotoxic drugs. Based on the well-established threshold of toxicological concern (TTC) concept, we defined an acceptable daily intake (ADI) of 4 μg/day as threshold of no concern for the risk assessment of occupational exposure to genotoxic drugs (excluding metals, proteins and substances with structural alerts for high potency carcinogenicity). We identified the dermal exposure to be the most relevant route of exposure of health workers to genotoxic drugs and estimated the total daily dermal intake (DDI) to be below 3.5 μg/day. Considering ADI and DDI, we derived an acceptable surface contamination level (ASCL) of 20 ng/cm^2^ for contaminated surfaces in healthcare facilities. A review of current studies on the drug contamination of surfaces in healthcare facilities showed that the ASCL is not exceeded in the majority of modern healthcare settings using appropriate protective equipment and cleaning protocols. Health workers in modern healthcare facilities are presumably not at risk of developing work related cancer due to the handling of genotoxic drugs.

An ADI of 4 μg/day was identified to protect health workers from developing work related cancer (lifetime excess cancer risk of 1:100.000). The simple mathematical models underlying this concept may readily be modified to fit regional regulatory requirements. For example, the German occupational safety and health legislation uses a tiered approach for setting risk-based occupational exposure levels (OELs) for carcinogens when a full toxicity database is available (see TRGS 910) [39]. According to TRGS 910, safety measures should aim on reducing workplace exposures to carcinogens to exposures that would not lead to an excess cancer risk of 4:100.000 (level of acceptance). A second level of tolerance (excess cancer risk of 4:1000) must not be exceeded. The level of tolerance relates to the statistical risk of a farm workers in Germany to be involved in a fatal accident during 40 working-years. Transferred to the exposure of health workers to genotoxic drugs, daily intakes of 16 μg/day (level of acceptance) and 1600 μg/day (level of tolerance) must not be exceeded. The corresponding ASCL would be 80 ng/cm^2^ (level of acceptance) and 8000 ng/cm^2^ (level of tolerance). This example demonstrates, that compared to our conservative risk assessment approach, the application of regional regulatory requirements could lead to the acceptance of even higher exposures.

Common antineoplastic drugs possess low vapour pressures [43]. They are handled using technical safety measures such as BSC to prevent inhalative exposure [4]. According to a recent study of Crul et al. (2020), dermal exposure can be effectively minimized by wearing suitable protective gloves [44]. These findings are supported by the results of our review indicating that the dermal contact to contaminated surfaces is considered to be the primary route of exposure of health workers to hazardous drugs. This is of particular interest, since existing risk assessment strategies from the pharmaceutical industry are usually focused on the control of inhalative exposure of workers to hazardous drugs [9]. It has to be noted, that our compilation of (dermal) exposure data is based on a short review only. It was designed to serve as a starting point for the risk assessment and does not include all available resources on drug exposure in healthcare settings. Additional exposure data will be necessary during evaluation of exposure scenarios not covered by our recent review (see Table 1).

We continued our TTC-based risk assessment under the assumption, that the dermal exposure is the most relevant route of exposure in healthcare settings and derived an ASCL of 20 ng/cm^2^ that sufficiently protects health workers from developing work related cancer. The average contamination of surfaces in modern healthcare facilities do not exceed the ASCL of 20 ng/cm^2^. This demonstrates that efforts to improve the occupational safety of health workers already have led to working conditions that are sufficiently protective.

It has to be noted, that the TTC-concept is a theoretical approach that does not represent a realistic indication of an actual risk [10]. In addition to that, the TTC-approach is not universally applicable to all drug molecules regardless of their chemical structure. Substances triggering structural alerts for high carcinogenic potency as well as metals and proteins cannot be assessed. Our review of chemical structures of common antineoplastic drugs showed, that these exclusion criteria are met by several active ingredients. Nevertheless, the vast majority of antineoplastic drug molecules is covered by the current risk assessment approach using the TTC-concept. Other drug molecules not included in our review of chemical structures have to be checked for the presence of the exclusion criteria on a case-by-case basis prior to the application of the TTC-based risk assessment approach. Data on the carcinogenic potency of (antineoplastic) drug molecules have not been systematically reviewed yet and/or are often not (publically) available. Future studies should address this issue. It may be warranted to exclude some potent carcinogens such as melphalan or mitomycin c by forming a specific cohort of concern for these drugs in addition to the consideration of the general exclusion criteria of the TTC-approach.

Refinements of the present approach will also have to evaluate the quantitative usage of individual drug substances. High volume APIs such as cyclophosphamide will probably have a different impact on the cancer risk then rarely handled high potency carcinogens.

In the absence of human data on the health effects of low dose multiple drug exposure, however, the TTC-concept is well suited to provide an estimate of the actual risks of health workers handling genotoxic drugs. The conservative assumptions made in this work during the adaption of the TTC-concept and the exposure assessment will rather over- than underestimate the actual risk and exposure of health workers. For example, we chose an ADI of 1.5 μg/day (single substance exposure) as point of departure (POD) for our risk assessment, although the ICH M7 guideline uses an ADI of 5 μg/day as POD for simultaneous exposures to multiple genotoxic substances [10]. It is furthermore likely, that the dermal exposure of health workers is overestimated in our model. Health workers usually use protective gloves during drug handling. Contact of the unprotected skin to contaminated surfaces usually occurs accidental and to a small degree (contamination of the fingertips). By assuming regular contact to larger skin areas (palms) and 100% uptake as suggested by Kimmel and co-workers, we used a worst-case scenario to estimate the dermal exposure and drug intake of health workers [13].

The present study focused on the adaption of the TTC-concept to develop a quantitative risk assessment tool for the exposure of health workers to genotoxic drugs. Future studies should aim on refining the dataset on the (dermal) exposure of health workers and the bioavailability of drugs following dermal exposure. Further limitations of this study include the fact that the risk assessment focusses solely on the evaluation of genotoxic properties of drugs. Other chemical hazards (such as irritating and sensitizing properties) have to be evaluated separately.

## Conclusions

Carcinogenic risks remain a major concern in ensuring the occupational safety of drug handling health workers. Using our TTC based risk assessment model, there is no evidence that acceptable risks levels for developing work related cancer from occupational exposure to genotoxic drugs are exceeded in modern healthcare settings. Hence, the present study may assist employers and public authorities to make informed decisions concerning the need for (further) protective measures and during risk communication to health workers.

## Data Availability

The datasets used and/or analysed during the current study are available from the corresponding author on reasonable request.

## Abbreviations

5-FU: 5-fluorouracil
ADI: Acceptable daily intake
AGS: Committee on Hazardous Substances (*german*)
API: Active pharmaceutical ingredient
ASCL: Acceptable surface contamination level
BSC: Biologic safety cabinet
CP: Cyclophosphamide
CSTD: Closed system drug transfer system
DDI: Daily dermal intake
DX: Docetaxel
EMA: European Medicines Agency
GM: Gemcitabine
IARC: International Agency for Research on Cancer
ICH: International Council for Harmonisation of Technical Requirements for Registration of Pharmaceuticals for Human Use
IF: Ifosphamide
MLI: Maximum lifetime intake
MT: Methotrexate
NIOSH: National Institute for Occupational Safety and Health
PPE: Personal protective equipment
Pt: Platinum compounds
PX: Paclitaxel
TRGS: Technical Rule for Hazardous Substances (*german*)
TTC: Threshold of toxicological concern
